# Determination of Polycyclic Aromatic Hydrocarbon Content in Garden Herbal Plants Using Liquid Chromatographic Analysis (HPLC-FL)

**DOI:** 10.3390/plants12030551

**Published:** 2023-01-25

**Authors:** Magdalena Woźniak, Karolina Hoppe, Kinga Drzewiecka

**Affiliations:** Department of Chemistry, Faculty of Forestry and Wood Technology, Poznań University of Life Sciences, Wojska Polskiego 75, 60625 Poznań, Poland

**Keywords:** PAH contaminants, HPLC, lavender, parsley, mint

## Abstract

Polycyclic aromatic hydrocarbons (PAHs) are a group of chemical compounds generated as a result of the incomplete combustion of fossil fuels or wood. PAHs are known for their negative effect on living organisms, including teratogenic, carcinogenic and mutagenic activity. The objective of this study is to determine the contamination of three popular herbal species showing pro-health properties, i.e., lavender, parsley and mint, with polycyclic aromatic hydrocarbons, collected from three different backyard gardens in Poland. The concentration of PAHs in plant material was determined by high-performance liquid chromatography with a fluorescence detector (HPLC-FL). The concentration of eleven PAHs in plant material was determined with high-pressure liquid chromatography after extraction using the QuEChERS purification technique. Mint collected within an area of a mining and energy production complex (the city of Konin) was characterized by the highest Σ of 11 PAHs, equaled to 902.35 µg/g FW, with anthracene being the most abundant compound. However, it contained the lowest sum of PAHs, among all tested plants, with high carcinogenicity. Parsley from the city of Poznań showed the highest content of benzo[a]pyrene (BaP), showing the strongest carcinogenicity, while the highest value of BaP equivalent was calculated for mint collected in Konin. The obtained results suggest that the level and profile of plant contamination with PAHs depend on the species and the location of herb cultivation. In particular, mining and energy industry facilities are sources of PAHs, which contaminate plant material for further direct use or as bioactive herbal extracts.

## 1. Introduction

Polycyclic aromatic hydrocarbons (PAHs) are a group of chemical compounds containing two to seven fused aromatic rings, which can be obtained from the incomplete combustion of fossil fuels or wood [1,2]. Particular PAHs are known for their negative effect on living organisms, including teratogenic, mutagenic and carcinogenic activity [3,4]. Moreover, they are characterized by low solubility in water, high lipophilicity and high mobility in the environment, which could pose a serious threat to the health of living organisms as well as ecosystems [5]. Among polycyclic aromatic hydrocarbons, naphthalene, fluorene, pyrene, anthracene, benzo[b]fluoranthene, benzo[a]pyrene, chrysene and acenaphthene have been enlisted as priority contaminants by the US Environmental Protection Agency (US EPA) and the European Union (EU) [6]. The presence of PAHs has been confirmed in soil, water, air, sediment and food [1,2]. Literature data indicate that plants can be recognized as bioindicators of environmental pollution with polycyclic aromatic hydrocarbons [3,5]. Moreover, various plant species exhibit significant differences in their capacity to accumulate PAHs [3].

The members of *Lavandula* genus include 47 species, along with infraspecific taxa and hybrids, with *Lavendula angustifolia* Mill. (English lavender) from the group of mints being the most widely cultivated species. *Lavendula* is native to the Canary Islands and distributed mainly in the Mediterranean zone, South West Asia, India and tropical Africa. All of them are rich in phenolic compounds, represented mainly by flavones hypolaetin and scutellarein in the form of glycosides, triterpenoids (including ursolic acid) and anthocyanins as floral pigments. Phenolic acid esters are present in leaves, while coumarin is present in essential oils [7]. Commercially, *Lavendula angustifolia* Mill. and its hybrids, called lavandins, are cultivated mainly for the production of scented essential oils and, to some extent, as ornamental plants. About 100 compounds have been identified as the ingredients of lavender oil, mainly linalyl acetated, linalool, tannins and caryophyllene, while a high level of terpenes, including camphor, is considered to lower the oil quality [8,9,10]. Besides essential oil production, dried lavender flowers (buds) are used in cooking to amplify sweet or savory flavor and to scent sugar and sweets, including chocolate or milk products, and the green parts are used as aromatic tea additives. Lavender flowers are also a rich source of pollen for bees to produce a monofloral aromatic lavender honey [10]. Lavender oil is commonly applied in aromatherapy to treat stress, anxiety or (postpartum) depression due to the ability of linalool to affect aminobutyric acid receptors in the central nervous system [11,12,13]. A significant decrease in cortisol release and an increased secretion of serotonin, along with a significant reduction of anxiety, during delivery after the inhalation of lavender was recently reported [14]. Other studies have also suggested the anti-aging, neuroprotective, antibacterial and even anticancer properties of lavender essential oil [15,16]. 

Parsley (*Petroselinum crispum* Mill.), of the family *Apiaceae*, is a native species to the central and eastern Mediterranean region; however, it is cultivated across Europe. The species is mostly used as a root vegetable, while edible leaves serve as an aromatic culinary herb added to improve the taste and enhance the flavor of food. All parsley plant parts (leaf, stem and root) are rich sources of phytonutrients with mainly antioxidant properties, including flavonoids (with the main flavones apigenin, chrysin and luteolin), vitamins (C and E), minerals (potassium, phosphorus, calcium, magnesium), and also chlorophylls and volatile oil compounds present in leaves or seeds (mainly α-pinene, β-pinene, myrcene, β-phellandrene and myristicin) [17,18]. Parsley is one of the world’s seven most potent disease-fighting spices [19]. The pharmacological activities of parsley include diuretic, hepato- and gastroprotective, brain-protective, antidiabetic, analgesic, immunosuppressant, cytoprotective, hypotensive, antibacterial and antifungal properties assayed for extracts and essential oils [20]. Among others, parsley presents a therapeutic effect on immune-mediated diseases such as allergy, autoimmune and chronic inflammatory disorders due to the immunomodulatory activity of its bioactive constituents [19]. 

Mint (*Mentha* spp.) is a popular culinary herb used in food and beverage products, mostly for its aroma and flavor. Genus *Mentha* of the mint family (*Lamiaceae*) comprises of 25 to 30 perennial species growing naturally in the northern USA and Canada and cultivated globally for their aromatic qualities and strong medicinal benefits [21]. Essential oils of mint plants are utilized in numerous applications, from fragrance and flavoring additives in cosmetics and spice mixtures to a component of bio-repellents or pesticides. The medicinal effects of mint result from its pro-digestive, antiseptic, antispasmodic, antirheumatic, expectorant or antitussive, antiallergic and antioxidant activities [21,22]. The main bioactive ingredients of mint oil are limonene, cineole, menthone, menthofuran, isomenthone, menthyl acetate, isopulegol, menthol, pulegone and carvone [23]. Flavonoid glycosides, phenolic acids and polyphenols of strong antioxidant activity are also present in the aerial parts of mint plants and can be extracted with a range of solvents. Among the phenolics, the highest contents were reported for narirutin, narigenin-7-oglucoside, luteolin-7-o-rutinoside, luteolin-diglucoronide, hesperidin, isorhoifolin, eriocitrin; rosmaric, cinnamic and caffeic acids [24,25]. 

The aim of the study is to determine the contamination of three popular herbal species showing significant pro-health properties, i.e., lavender, parsley and mint, commonly used as culinary herbs, with polycyclic aromatic hydrocarbons. Since the selected species are widely cultivated in backyard gardens, three sampling sites with different characteristics of air pollution in Poland were chosen for the study. In plant material, the content of eleven PAHs was determined by high-pressure liquid chromatography (HPLC). 

## 2. Results and Discussion 

The concentration of eleven compounds from polycyclic aromatic hydrocarbons (fluoranthene, fluorene, acenaphthene, pyrene, benzo[a]anthracene, benzo[a]pyrene, anthracene, benzo[b]fluoranthene, phenanthrene, naphthalene and acenaphthylene) was determined in three popular garden plants, namely, lavender, parsley and mint, and the results are presented in Table 1. The PAH content in the plant material was calculated on fresh weight because parsley and mint leaves are normally consumed as fresh herbs. 

All the determined polycyclic aromatic hydrocarbons are listed as a priority by the US EPA and the EU due to their proven toxic and carcinogenic properties. The results presented in Table 1 indicate that all tested plant species contained PAHs, which demonstrates the widespread nature and persistence of the assayed compounds. The examined plants were characterized by varied concentrations of polycyclic aromatic hydrocarbons, both qualitatively and quantitatively. Anthracene (ANT) was found to be the most abundant compound in the analyzed plant material. The highest concentration of anthracene (207 µg/g FW) was detected in mint from the first location (Konin). Fluorene (FL) was the only compound detected in all examined plant samples, in a concentration range from 352 (mint, Site 3) to 28,129 ng/g FW (parsley, Site 2). Acenaphthylene (ACY) was detected only in four among nine plant samples, in a range from 1761.73 (mint, Site 2) to 18,802.75 ng/g FW (parsley, Site 1), and was not presented in any lavender samples. In turn, the presence of phenanthrene (PHE) was confirmed only in three samples—mint (2046 ng/g FW) and lavender (10,826 ng/g FW) collected in Konin (Site 1), and parsley (10,127 ng/g FW) from Poznań (Site 3). A high concentration of acenaphthene (ACE) was determined in five plant samples, ranging from 9287 ng/g FW (lavender, Site 2) to 67,692 ng/g FW (mint, Site 1). Moreover, high contents of naphthalene (NP; in a concentration range of 435–611,196 ng/g FW) and anthracene (ANT; in a range of 7736–207,022 ng/g FW) were also confirmed in the investigated plant material. 

An analysis of all determined PAH content indicated that mint from Site 1 was characterized by the highest Σ of 11 PAHs, equaled to 902 µg/g FW, while mint from Site 3 contained the lowest Σ of 11 PAHs of 9.13 µg/g FW (Table 1). A high content of all examined compounds of 221 µg/g FW was also detected in parsley collected from the first location. The high PAH contamination of plants from the first location may be related to the presence of a lignite mine and a complex of three energy plants in this area. 

**Figure 1:**
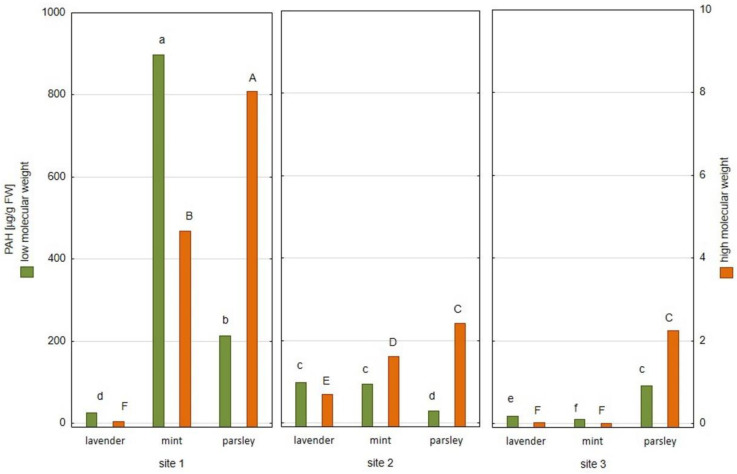
The content of low and high molecular weight PAHs expressed as average (*n* = 3) (values denoted with identical letters do not differ significantly at *p* = 0.05 according to the post-hoc test, following two-way ANOVA for the ‘site×species’ fixed effect; FW—fresh weight).

The sums of low molecular weight PAHs (compounds with three or less rings) and high molecular weight PAHs (compounds with four or more rings) determined in the plant material are presented in Figure 1. The highest sum of low molecular weight PAHs (including acenaphthene, fluorene, acenaphthylene, naphthalene, phenanthrene and anthracene) was determined in mint from Site 1 (897.67 µg/g FW), and the lowest was detected in mint collected from Site 3 (9.13 µg/g FW). In turn, parsley from Site 1 was characterized by the highest sum of high molecular weight PAHs (including fluoranthene, pyrene, benzo[a]anthracene, benzo[a]pyrene and benzo[b]fluoranthene), equaled to 8.08 µg/g FW, while mint from Site 3 contained the lowest total sum of high molecular weight PAHs (1.98 ng/g FW). Moreover, parsley samples collected from all three locations were characterized by the highest sum of high molecular weight PAHs compared to other plant samples collected from the same locations (lavender and mint). The low molecular weight PAHs are less lipophilic, more volatile, and more water-soluble than the high molecular weight PAHs. Moreover, high molecular weight PAHs are recognized as human carcinogens [26,27,28]. 

**Figure 2:**
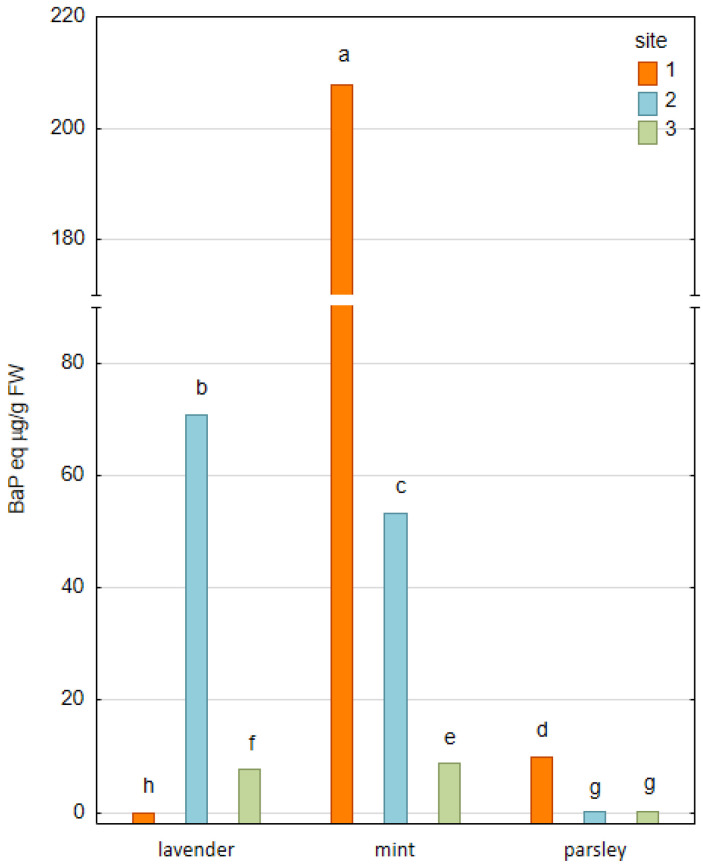
The content of benzo[a]pyrene equivalent (BaP_eq_) in plant material expressed as average (*n* = 3) (values denoted with identical letters do not differ significantly at *p* = 0.05 according to the post-hoc test, following two-way ANOVA for the ‘site×species’ fixed effect; FW—fresh weight).

Among over 400 carcinogenic PAHs and their derivates, benzo[a]pyrene (BaP) shows the strongest carcinogenicity [29,30]. It is a five-ring polycyclic aromatic hydrocarbon with a negative impact on human health, including teratogenicity, tumor formation, immunosuppression, osteotoxicity and hormonal effects [31,32,33]. The highest content of BaP was detected in parsley from Site 3 (46.53 ng/g FW), while its content in parsley collected from Site 2 was under the detection limit of the applied chromatographic method. In the literature data, the BaP equivalent (BaP_eq_) is often used to assess the toxicity of the profiled PAH contamination. The BaP_eq_, calculated based on the Agency for Toxic Substances and Disease Registry’s (ATSDR’s) approach [34], in plant material is presented in Figure 2. The highest value of BaP_eq_ was obtained for mint collected at Site 1 (207.73 µg/g FW), while the lowest value was described in lavender from the same site (25.42 ng/g FW). 

**Figure 3:**
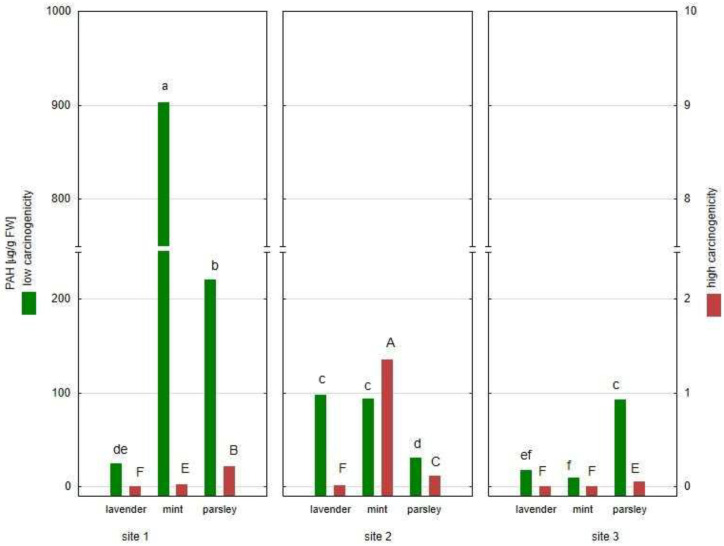
The content of low and high carcinogenicity PAHs in plant material expressed as average (*n* = 3) (values denoted with identical letters do not differ significantly at *p* = 0.05 according to the post-hoc test, following two-way ANOVA for the ‘site×species’ fixed effect; FW—fresh weight).

The US EPA has classified 16 polycyclic aromatic hydrocarbons as carcinogenic, including seven compounds with probable high carcinogenicity (benzo[a]anthracene, benzo(b)fluoranthene, benzo(k)fluoranthene, benzo(a)pyrene, indeno(123 cd)pyrene, dibenzo(ah)anthracene and benzo(ghi)pyrelene) [35]. The contents of high carcinogenicity PAHs (benzo[a]anthracene, benzo(b)fluoranthene and benzo(a)pyrene) and low carcinogenicity PAHs (naphthalene, acenaphthene, acenaphthylene, fluorene, fluoranthene, pyrene, phenanthrene and anthracene) in the plant material are presented in Figure 3. 

The sum of PAHs with high carcinogenicity was in the range of 0.001 (mint, Site 3) to 1.354 µg/g FW (mint, Site 2). The results presented in Figure 3 showed that all examined plant material contained at least one of the highly carcinogenic PAHs. Moreover, four of the tested plants (lavender from Site 2, parsley from Site 1 and mint from sites 1 and 2) contained three compounds with high oncogenic activity. In turn, mint from Site 1 was characterized by the highest sum of low carcinogenicity PAHs (902.3 µg/g FW), while mint from Site 3 showed the lowest sum of low carcinogenicity PAHs (9.1 µg/g FW). In addition, parsley from Site 1 contained a high level of low carcinogenicity PAHs (220.5 µg/g FW). 

The diversity of compounds belonging to polycyclic aromatic hydrocarbons and their derivatives have been detected in a variety of plant materials, including medical plants, fruits, vegetables and spices [36,37,38,39]. The presence of four PAHs (benzo[a]pyrene, chrysene, benzo[b]fluoranthene and benzo[a]anthracene) in 86% of 150 dried herbs samples (including thyme, oregano, basil, black pepper, nutmeg and paprika) was confirmed by Rozentale et al. [40]. The PAH content in parsley samples collected from urban and rural areas in Romania was assessed by Soceanu et al. [38]. The authors found that parsley from urban areas contained higher contents of the examined PAHs than parsley from rural areas and that parsley leaves were characterized by a higher content of PAHs than its stems. In turn, parsley collected in the Czech Republic contained higher contents of PAHs than cucumber, tomato or apple harvested from the same area. Parsley was characterized by a high content of phenanthrene (3.77–5.55 µg/kg fresh mass) and 1-methylnaphthalene (4.41–5.33 µg/kg fresh mass). Wennrich et al. [36] examined the PAH concentrations in fruit and vegetable species and found that parsley and kale were characterized by the highest PAH content. Parsley from industrial areas of Germany contained high levels of phenanthrene (8.48–73.86 µg/kg fresh weight) and fluoranthene (2.07–14.26 µg/kg fresh weight) [36]. Krajian and Odeh [41] analyzed the content of 16 US EPA PAHs in ten medical plants from Syria and indicated that the highest contents of the examined PAHs were found in the sage plant sample. In turn, Ishizaki et al. [42] examined PAH contents in tea and herb samples and showed that the mint herb contained a high content of phenanthrene (23.1 ng/g), pyrene (19.4 ng/g) and fluoranthene (22.5 ng/g). The presence of PAHs in mint tea samples from the Polish market was confirmed by Ciemniak et al. [43], with high content of phenanthrene (49.9 µg/kg) and fluoranthene (39.0 µg/kg). 

Tao et al. [44] examined PAH contents in several types of vegetables (including cabbage, celery, spinach, carrot and cauliflower) and found that the concentration of 16 PAHs in the aerial part of vegetables was 6.5 times higher than that in roots, which suggested that foliar uptake was the primary transfer pathway of PAHs from the environment to vegetables. In turn, Ashraf et al. [45] analyzed PAH contents in vegetables (carrot, cabbage, potato, tomato, cucumber, spinach) from Saudi Arabia and indicated that leafy vegetables (spinach and cabbage) contained high levels of PAHs. The authors found that the adsorption of PAHs from the air was significantly higher in spinach and cabbage due to their large leaf surface [45]. 

Air polluted with polycyclic aromatic hydrocarbons may be the main source of PAH contamination in the tested plant materials. According to a report entitled “Air pollution with polycyclic aromatic hydrocarbons at urban background stations in Poland in 2017”, prepared by the Chief Inspectorate for Environmental Protection, the concentrations of polycyclic aromatic hydrocarbons, including B(a)P, are much higher in Poland than in most European countries [46]. According to the report, the most significant amounts of PAHs (88%) are emitted from solid fuel boilers and stoves used to heat buildings. The average concentration of the three PAHs analyzed in the study (BaP, BaA and BbF), determined in the air in the Wielkopolskie Province (material samples collected in Konin and Poznań) and from the Pomeranian Province (samples collected in Tczew), are presented in the report [46]. The lavender and mint collected from Tczew (Site 2) were characterized by higher BaP content than in plants from other gardens. In turn, the BaP concentration in parsley from Tczew was under the detection limit of the HPLC method, while parsley from Poznań (Site 3) contained a very high content of the same (46.53 ng/g FW). The average content of BaP in the Pomeranian Province was 4.29 ng/m^3^, while in the Wielkopolskie Province, it was 4.02 ng/m^3^; in both cases, it was higher than the target level (1.5 ng/m^3^). The contents of BaA and BbF in the Pomeranian Province equaled 0.65 and 1.24 ng/m^3^, while in the Wielkopolska Province, the content of these compounds reached 3.11 and 3.16 ng/m^3^, respectively [46]. Lavender and mint from Site 2 (Tczew) contained a higher content of BaA than herbs from other sites. Moreover, parsley from this site was characterized by a lower BaA content than plant material from Konin, and the concentration of this PAH in parsley from Poznań was under the detection limit of HPLC determination. A higher content of BbF was found in lavender from Tczew (Site 2) and mint and horse parsley (Site 1). It seems that the concentration of individual PAHs in the tested plant material depends on the species and the place of collection. No relationship was observed between the concentration of the analyzed compounds in the air and in the plant, which may be related to the fact that the measuring stations were not located exactly in the same places from which the research material was collected. Further research is required to determine the factors that provided the PAH content in the tested garden plants.

## 3. Materials and Methods

### 3.1. Plant Material

**Figure 4:**
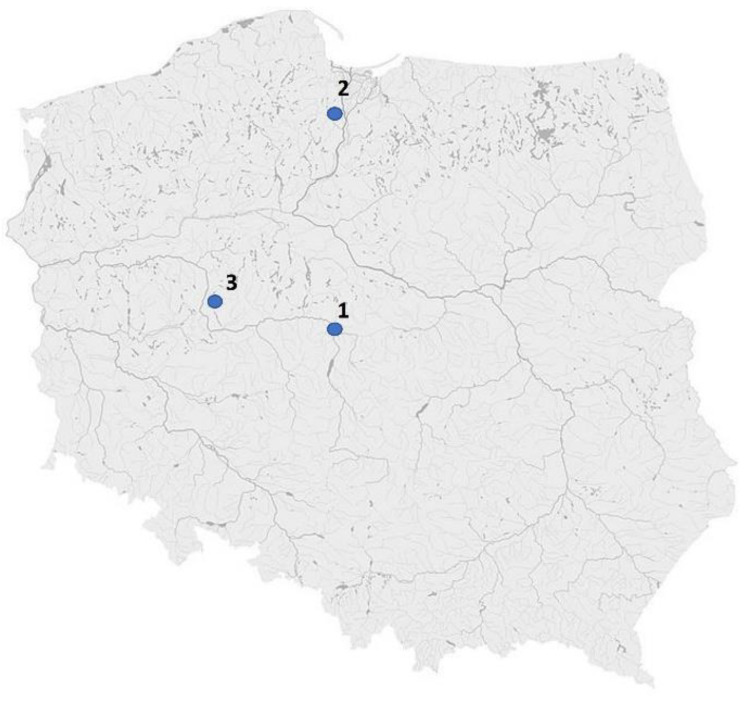
The sites of plant collection in Poland (1—Konin, 2—Tczew, 3—Poznań).

In the study, three popular Polish garden plants were used, namely, *Lavandula angustifolia* Mill. (variety Blue Scent), *Mentha* x *piperita* L. (variety Chocolate) and *Petroselinum crispum* Mill. Fuss. (variety Gigante D’Italia). All plant material was purchased from local stores (Sadowniczy, Kadzidło, Poland) and planted in three gardens in different regions of Poland at the same time. The herbs were cultivated in gardens located in three cities in Poland: Konin (1), Tczew (2) and Poznań (3) (Figure 4). Site 1 was a garden placed on the outskirts of Konin, within the Konin Brown Coal Basin and a lignite mine and energy plant complex. Moreover, plant samples were collected from home gardens in the center of Tczew (Site 2) and gardens located on the outskirts of Poznań (Site 3), in a place with little traffic and a motorway about 800 meters away. The plant material was collected from the gardens in the summer of 2022, in the second year after planting all the plants in the anthesis vegetation stage. Lavender buds, including young stems with flowers, as well as shoots and leaves of mint and parsley, were used for the analysis. 

### 3.2. PAH Standards

The standards of PAHs (fluoranthene (FLA), fluorene (FL), acenaphthene (ACE), pyrene (PYR), benzo[a]anthracene (BaA), benzo[a]pyrene (BaP), anthracene (ANT), benzo[b]fluoranthene (BbF), phenanthrene (PHE), naphthalene (NP), acenaphthylene (ACY)) were dissolved in a mixture of acetonitrile and methanol (60:40; v/v) to obtain a final concentration of each compound of 1 mg/mL. The glassware was washed and rinsed with ultrapure solvents (acetone and hexane) before use.

### 3.3. PAH Extraction from Plant Material

PAHs were extracted from plant material by the QuEChERS purification technique. The QuEChERS columns (SupelMIP SPE-PAHs) were purchased from Sigma Aldrich (Darmstadt, Germany). Plant samples (1 g of fresh weight) were placed in 50 mL centrifuge tubes and extracted with hexane (3 mL) using a laboratory shaker (Biosan, Riga, Latvia) for 24 h at an ambient temperature. After centrifugation (5 min, 3000 rpm; Universal 320, Andreas Hettich GmbH and Co. KG, Tuttlingen, Germany), the extract (1.5 mL) was transferred onto a QuEChERS column. The column was conditioned with hexane (1 mL) before the mixture was applied. Hexane (1 mL) was used to wash the extract, followed by three times elution with ethyl acetate (1 mL). SPE eluates were evaporated under a nitrogen atmosphere at 40 °C, reconstituted in 0.5 mL of a mixture of acetonitrile and methanol (60:40; v/v) and filtered through a 0.20 µm syringe filter (Chromafil, Macherey-Nagel, Duren, Germany) before chromatographic analysis.

### 3.4. PAH Determination by High-Pressure Liquid Chromatography

The concentration of particular PAHs was determined using a chromatographic system: a Waters 2695 high-performance liquid chromatograph (Waters, Manchester, MA, USA) and a Waters 2475 Multi-λ fluorescence detector (Waters, Manchester, MA, USA). PAH determination was carried out using a fluorescence detector and the excitation and emission wavelengths of 300 and 466 nm, respectively. For PAH separation, a Pursuit PAH (1250 × 4.6 mm; particle size 5 μm) column (Agilent Technologies, Santa Clara, CA, USA) and a Pursuit PAH safeguard column (Agilent Technologies, Santa Clara, CA, USA) were used. The mobile phase was acetonitrile:water at a flow rate of 0.5 mL/min. The mobile phase gradient is presented in Table 2. 

The injection volume and column temperature were 5.0–20 μL and 22 °C, respectively. A standard curve was established using the peak of the eleven PAHs, which corresponded to their concentrations (0–200 μg/g, correlation coefficient ≥ 0.9977, Table 3). The limit of detection and limit of quantitation of particular PAHs were calculated using the signal-to-noise ratios of 3 and 10 and were 0.03–0.26 and 0.10–0.88 ng/g, respectively. 

## 4. Conclusions

The paper presents the results of the contamination of three popular herbal species showing significant pro-health properties, i.e., lavender, parsley and mint, with eleven polycyclic aromatic hydrocarbons, listed as a priority by the US EPA and the EU due to their proven toxic and carcinogenic nature.

The examined plants were characterized by varied concentrations of polycyclic aromatic hydrocarbons, both qualitatively and quantitatively. Anthracene was found to be the most abundant compound in the analyzed plant materials, with the highest concentration of 207.0 µg/g FW in mint. The analysis of all determined PAH content indicated that mint from Site 1 (Konin) was characterized by the highest Σ of 11 PAHs of 902.35 µg/g FW, while mint from Site 3 (Poznań) contained the lowest Σ of 11 PAHs, equal to 9.13 µg/g FW. The mint from Site 1 was characterized by the highest sum of low molecular weight PAHs (897.67 µg/g FW), while parsley (also Site 1) was characterized by the highest sum of high molecular weight PAHs (8.08 µg/g FW). In turn, mint from Site 3 contained the lowest sum of both low and high molecular weight PAHs. The high PAH contamination of plants from the first location may be related to the presence of a lignite mine and a complex of three energy plants in this area. Parsley from Site 3 showed the highest content of benzo[a]pyrene (BaP), which shows the strongest carcinogenicity. However, the highest value of BaP_eq_ was calculated for mint from Site 1 (207.73 µg/g FW). The sum of PAHs with high carcinogenicity was in the range of 0.001 (mint, Site 1) to 1.354 µg/g FW (mint, Site 2). In turn, mint from Site 1 was characterized by the highest sum of low carcinogenicity PAHs (902.3 µg/g FW), while mint from Site 3 showed the lowest sum of low carcinogenicity PAHs (9.1 µg/g FW). 

The obtained results indicate that popular garden herbs are contaminated with polycyclic aromatic hydrocarbons. The qualitative and quantitative contamination of the plant material depends on the plant species and the location of the garden from which the material is collected.

## Figures and Tables

**Table 1:** The concentration of PAHs in aerial parts of plants (buds of lavender, shoots and leaves of mint and parsley) collected in three locations (FLA—fluoranthene, FL—fluorene, ACE—acenaphthene, PYR—pyrene, BaA—benzo[a]anthracene, BaP—benzo[a]pyrene, ANT—anthracene, BbF—benzo[b]fluoranthene, PHE—phenanthrene, NP—naphthalene, ACY—acenaphthylene).

PAH	Content (ng/g FW)								
	Lavender			Mint			Parsley		
	Site								
	1	2	3	1	2	3	1	2	3
**FLA**	33.30 ^f^ ± 0.01	76.11 ^e^ ± 0.75	2.67 ^g^ ± 0.02	442.75 ^b^ ± 5.74	6.31 ^g^± 0.01	nd	92.56 ^d^ ± 1.76	2295.43 ^a^ ± 4.26	144.33 ^c^ ± 2.39
**FL**	433.49 ^f,g^ ± 0.43	1998.48 ^e^ ± 6.96	7999.72 ^c^ ± 10.28	6340 ± 44 ^d^ ± 4.10	682.36 ^f^ ± 1.61	352.15 ^g^ ± 1.77	1749.19 ^e^ ± 8.37	28,129.41 ^a^ ± 12.01	10,032.30 ^b^ ± 16.46
**ACE**	nd	9286.69 ^e^ ± 8.38	nd	67,692.36 ^a^ ± 4.92	25,296.59 ^d^ ± 4.76	nd	27,695.09 ^c^ ± 67.79	nd	58,662.85 ^b^ ± 49.06
**PYR**	nd	590.91 ^d^ ± 7.39	nd	4209.67 ^b^ ± 3.67	241.19 ^e^ ± 3.19	nd	7780.78 ^a^ ± 17.05	nd	2048.47 ^c^ ± 8.52
**BaA**	nd	8.74 ^d^ ± 0.09	nd	11.77 ^d^ ± 0.24	1336.48 ^a^ ± 2.38	nd	170.75 ^b^ ± 3.85	111.52 ^c^ ± 1.69	nd
**BaP**	0.91 ^f,g^ ± 0.01	4.65 ^c,d^ ± 1.35	6.03 ^c^± 0.07	2.75 ^e^ ± 0.04	14.33 ^b^ ± 0.27	1.98 ^e,f^ ± 0.01	3.25 ^d,e^ ± 0.08	nd	46.53 ^a^ ± 0.72
**ANT**	nd	70,782.40 ^b^ ± 9.70	7736.28 ^f^ ± 24.50	207,022.05 ^a^ ± 69.48	51,861.66 ^c^ ± 48.77	8779.67 ^e^ ± 7.95	9408.95 ^d^ ± 4.56	nd	nd
**BbF**	0.11 ^g^± 0.01	1.74 ^e^ ± 0.01	0.27 ^f^ ± 0.01	6.78 ^b^ ± 0.08	2.96 ^d^ ± 0.05	nd	36.24 ^a^ ± 0.91	1.65 ^e^ ± 0.04	3.32 ^c^± 0.03
**PHE**	10,825.64 ^a^ ± 2.86	nd	nd	2045.64 ^c^ ± 8.43	nd	nd	nd	nd	10,126.56 ^b^ ± 9.25
**NP**	13,241.99 ^e^ ± 5.51	14,785.33 ^c^ ± 3.73	1488.27 ^f^ ± 5.72	61,195.55 ^a^ ± 14.32	14,093.97 ^d^ ± 68.17	nd	15,497.61 ^b^ ± 7.64	nd	434.72 ^g^ ± 3.26
**ACY**	nd	nd	nd	3378.14 ^c^ ± 7.45	1761.73 ^d^ ± 7.74	nd	18,802.75 ^a^ ± 17.58	nd	11,145.45 ^b^ ± 8.25
**Σ 11 PAHs**	24,501.4 ^g^	97,535.0 ^c^	17,233.2 ^h^	902,347.9 ^a^	95,297.6 ^d^	9133.8 ^i^	220,719.2 ^b^	30,538.0 ^f^	92,644.5 ^e^

Data presented as average (*n* = 3) ± standard deviation. Values denoted with identical letters do not differ significantly at *p* = 0.05 according to the post-hoc test, following two-way ANOVA for the ‘site×species’ fixed effect; FW—fresh weight, nd—not detected.

**Table 2:** Mobile phase gradient used in the HPLC analysis of PAHs.

Time (min)	% Acetonitrile	% Water
0	50	50
2	50	50
50	100	0
60	100	0
65	50	50

**Table 3:** Parameters of method validation (retention time, coefficients of linearity (r^2^), linearity, limit of detection (LOD) and limit of quantification (LOQ) for the HPLC-FL method).

PAHs	Retention Time (min)	Coefficients of Linearity (r^2^)	Linearity (µg/g)	LOD (ng/g)	LOQ (ng/g)
NP	12.90	0.9979	0–100	0.22	0.75
ACY	14.40	0.9981	0–20	0.26	0.88
ACE	16.73	0.9984	0–100	0.19	0.63
FL	17.04	0.9982	0–30	0.21	0.82
PHE	18.70	0.9999	0–20	0.19	0.65
ANT	20.10	0.9977	0–200	0.09	0.32
FLA	22.26	0.9985	0–10	0.18	0.62
PYR	23.15	0.9988	0–10	0.19	0.63
BaA	27.27	0.9998	0–5	0.05	0.17
BbF	32.48	0.9998	0–0.1	0.03	0.10
BaP	34.68	0.9999	0–0.1	0.20	0.67

## Data Availability

Not applicable.
